# 1,4-Bis[2-(1,3-benzothia­zol-2-yl)phen­oxy]butane

**DOI:** 10.1107/S1600536808002705

**Published:** 2008-01-30

**Authors:** Orhan Büyükgüngör, Arzu Özek, Senem Karahan, Elif Subaşı

**Affiliations:** a Department of Physics, Ondokuz Mayıs University, TR-55139 Samsun, Turkey; b Department of Physics, Dokuz Eylül University, İzmir, Turkey

## Abstract

The mol­ecule of the title compound, C_30_H_24_N_2_O_2_S_2_, adopts a *transoid* conformation consistent with the inversion centre located at the mid-point of the central C—C single bond, resulting in one half mol­ecule in the asymmetric unit. The dihedral angle between the coplanar benzothia­zole ring system and the benzene ring is 11.06 (7)°. In the crystal structure, mol­ecules are linked by weak inter­molecular π–π inter­actions between thia­zole and benzene rings to form a three-dimensional network.

## Related literature

For general background, see: Delmas *et al.* (2002 ▶); Karalı *et al.* (2004 ▶); Weinstock *et al.* (1987 ▶); Chopade *et al.* (2002 ▶); Di Nunno *et al.* (2000 ▶); Gökhan *et al.* (2004 ▶). For related structures, see: Sieroń *et al.* (1999 ▶); Usman *et al.* (2003 ▶). For related literature, see: Temel *et al.* (2008 ▶).
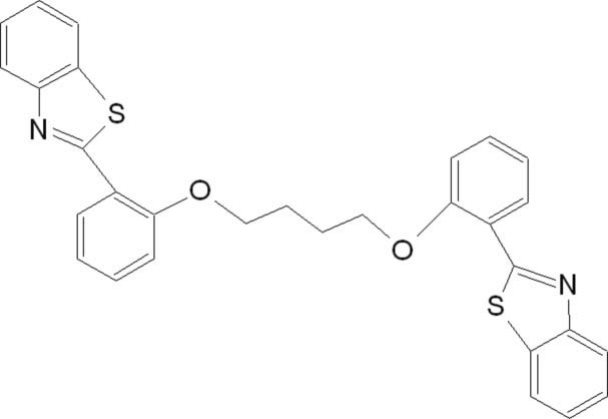


## Experimental

### 

#### Crystal data

C_30_H_24_N_2_O_2_S_2_
*M* *_r_* = 508.63
Monoclinic, 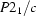

*a* = 14.3251 (13) Å
*b* = 4.8992 (3) Å
*c* = 17.4954 (17) Å
β = 102.522 (7)°
*V* = 1198.65 (18) Å^3^
*Z* = 2
Mo *K*α radiation
μ = 0.26 mm^−1^
*T* = 296 K
0.80 × 0.36 × 0.08 mm

#### Data collection

Stoe IPDSII diffractometer
Absorption correction: integration (*X-RED32*; Stoe & Cie, 2002 ▶) *T*_min_ = 0.442, *T*_max_ = 0.936
14397 measured reflections
2339 independent reflections
1456 reflections with *I* > 2σ(*I*)
*R*_int_ = 0.075

#### Refinement

*R*[*F* ^2^ > 2σ(*F* ^2^)] = 0.034
*wR*(*F* ^2^) = 0.069
*S* = 0.84
2339 reflections
163 parameters
H-atom parameters constrained
Δρ_max_ = 0.14 e Å^−3^
Δρ_min_ = −0.19 e Å^−3^

### 

Data collection: *X-AREA* (Stoe & Cie, 2002 ▶); cell refinement: *X-AREA*; data reduction: *X-RED32* (Stoe & Cie, 2002 ▶); program(s) used to solve structure: *SHELXS97* (Sheldrick, 2008 ▶); program(s) used to refine structure: *SHELXL97* (Sheldrick, 2008 ▶); molecular graphics: *ORTEP-3 for Windows* (Farrugia, 1997 ▶); software used to prepare material for publication: *WinGX* (Farrugia, 1999 ▶).

## Supplementary Material

Crystal structure: contains datablocks I, global. DOI: 10.1107/S1600536808002705/hk2423sup1.cif

Structure factors: contains datablocks I. DOI: 10.1107/S1600536808002705/hk2423Isup2.hkl

Additional supplementary materials:  crystallographic information; 3D view; checkCIF report

## Figures and Tables

**Table 1:** The observed π–π interaction distances (Å) for the title compound.

*Cg*—*Cg*^i^	*d*_centroids_	*d*_perpendicular_
*Cg*1—*Cg*2^i^	3.775 (11)	3.515
*Cg*1—*Cg*3^i^	3.7934 (12)	3.59

*Cg*1, *Cg*2 and *Cg*3 are the centroids of atoms S1/N1/C1/C6/C7, (C1–C6) and (C8–C13) rings, respectively. Symmetry code: (i) 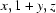.

**Fig. 1.**
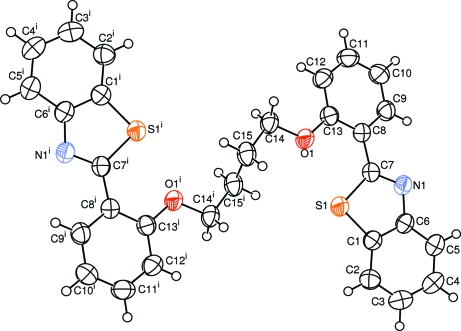
A view of (I) with the atom-numbering scheme. Displacement ellipsoids are drawn at the 30% probability level [symmetry code: (i) -x, -y, -z].

**Fig. 2.**
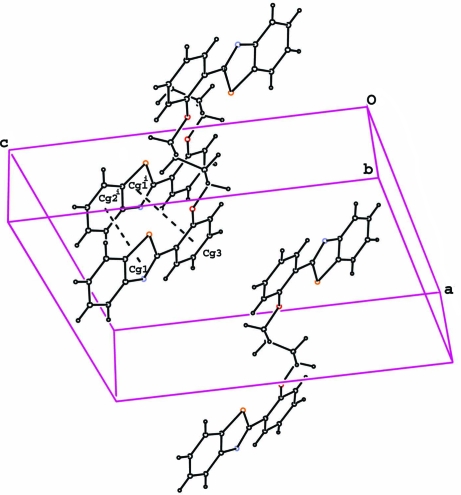
A partial packing diagram of (I), showing the π···π bonds (drawn as dashed lines) [symmetry code: (i) x, 1 + y, z].

## supplementary crystallographic information

### Comment

Benzothiazole derivatives possess a broad spectrum of pharmacological activity, including antibacterial, antifungal (Delmas *et al.*, 2002; Karalı *et al.*, 2004), dopaminergic (Weinstock *et al.*, 1987), anticonvulsant (Chopade *et al.*, 2002), antiadrenergic (Di Nunno *et al.*, 2000) and analgesic anti-inflammatory activities (Gökhan *et al.*, 2004). We report herein the synthesis and structure of the title compound, (I), which is a new benzothiazole derivative.

The molecule of the title compound, (I), (Fig. 1) displays an inversion centre with a half molecule in the asymmetric unit. The benzene ring and its fused thiazole ring are nearly coplanar, with the maximum deviation from the least-squares plane through S1/N1/C1—C7 occurring at S1 [0.033 (9) Å]. However, the molecule itself is nonplanar; the dihedral angle between the coplanar benzothiazole ring system and benzene ring is 11.06 (7)°. The N1—C7 [1.299 (2) Å] bond indicates double-bond character, whereas the S—C bond lengths are indicative of significant single-bond character. The S1—C1 [1.7231 (19) Å] bond is shorter than S1—C7 [1.7552 (18) Å], due to the fact that C7 is *sp*^2^ hybridized, whereas C1 is part of the aromatic ring. A similar effect was observed for *cis*-bis(2-amino-1,3-benzothiazole-*N*^3^)bis- (formato-O,*O*')copper(II) [(II); Sieroń *et al.*, 1999] and diacetatobis- (2-aminobenzothiazole)zinc(II) [(III); Usman *et al.*, 2003]. The corresponding N—C and S—C values are [N1—C2 = 1.321 (3) Å, S1—C2 = 1.742 (3) Å and S1—C8 = 1.741 (3) Å, in (II)] and [N1—C1 = 1.311 (3) Å, N3—C8 = 1.317 (3) Å, S1—C1 = 1.747 (2) Å, S1—C2 = 1.733 (3) Å, S2—C8 = 1.751 (2) Å and S2—C9 = 1.749 (3) Å, in (III)].

In the crystal structure, the molecules are linked by weak intermolecular π···π interactions (Table 1) between thiazole and benzene rings to form a three-dimensional network (Fig. 2).

### Experimental

The title compound, (I), was prepared by the literature method (Temel *et al.*, 2008). It was obtained from the photochemical reaction of *M*(CO)_5_ THF (*M*= Cr) (132 mg, 0.5 mmol) with *N*,*N*'-bis(2-aminothiophenol)-1,4-bis(2-carboxaldehyde- phenoxy) butane (153 mg, 0.3 mmol) in THF for 2 h at room temperature. UV irradiation was performed with a medium-pressure (125 W) mercury lamp through a quartz-walled immersion well reactor, which was cooled by circulating water. After the photochemical reaction, the solvent was removed under vacuum afford a solid residue which was dissolved in CH_2_Cl_2_ and then petroleum ether was added for the purification process. The solution was allowed to cool in a deep-freezer. Small colorless crystals grown in the CH_2_Cl_2_ /petroleum ether solution were filtered off and finally dried.

### Refinement

H atoms were positioned geometrically, with C—H = 0.93 and 0.97 Å for aromatic and methylene H, respectively, and constrained to ride on their parent atoms, with *U*_iso_(H) = 1.2*U*_eq_(C).

### Crystal data

**Table:**

C_30_H_24_N_2_O_2_S_2_	*F*_000_ = 532
*M**_r_* = 508.63	*D*_x_ = 1.409 Mg m^−^^3^
Monoclinic, *P*2_1_/*c*	Mo *K*α radiation λ = 0.71073 Å
Hall symbol: -P 2ybc	Cell parameters from 14397 reflections
*a* = 14.3251 (13) Å	θ = 1.7–28.0º
*b* = 4.8992 (3) Å	µ = 0.26 mm^−^^1^
*c* = 17.4954 (17) Å	*T* = 296 K
β = 102.522 (7)º	Thin long plate, colorless
*V* = 1198.65 (18) Å^3^	0.80 × 0.36 × 0.08 mm
*Z* = 2	

### Data collection

**Table:**

Stoe IPDSII diffractometer	2339 independent reflections
Radiation source: fine-focus sealed tube	1456 reflections with *I* > 2σ(*I*)
Monochromator: graphite	*R*_int_ = 0.075
Detector resolution: 6.67 pixels mm^-1^	θ_max_ = 26.0º
*T* = 296 K	θ_min_ = 2.4º
ω scans	*h* = −16→17
Absorption correction: integration(X-RED32; Stoe & Cie, 2002)	*k* = −6→6
*T*_min_ = 0.442, *T*_max_ = 0.936	*l* = −21→21
14397 measured reflections	

### Refinement

**Table:**

Refinement on *F*^2^	Secondary atom site location: difference Fourier map
Least-squares matrix: full	Hydrogen site location: inferred from neighbouring sites
*R*[*F*^2^ > 2σ(*F*^2^)] = 0.034	H-atom parameters constrained
*wR*(*F*^2^) = 0.069	*w* = 1/[σ^2^(*F*_o_^2^) + (0.0302*P*)^2^] where *P* = (*F*_o_^2^ + 2*F*_c_^2^)/3
*S* = 0.84	(Δ/σ)_max_ = 0.001
2339 reflections	Δρ_max_ = 0.14 e Å^−^^3^
163 parameters	Δρ_min_ = −0.19 e Å^−^^3^
Primary atom site location: structure-invariant direct methods	Extinction correction: none

### Special details

**Table:**

Experimental. 322 frames, detector distance = 100 mm
Geometry. All e.s.d.'s (except the e.s.d. in the dihedral angle between two l.s. planes) are estimated using the full covariance matrix. The cell e.s.d.'s are taken into account individually in the estimation of e.s.d.'s in distances, angles and torsion angles; correlations between e.s.d.'s in cell parameters are only used when they are defined by crystal symmetry. An approximate (isotropic) treatment of cell e.s.d.'s is used for estimating e.s.d.'s involving l.s. planes.
Refinement. Refinement of *F*^2^ against ALL reflections. The weighted *R*-factor *wR* and goodness of fit *S* are based on *F*^2^, conventional *R*-factors *R* are based on *F*, with *F* set to zero for negative *F*^2^. The threshold expression of *F*^2^ > σ(*F*^2^) is used only for calculating *R*-factors(gt) *etc*. and is not relevant to the choice of reflections for refinement. *R*-factors based on *F*^2^ are statistically about twice as large as those based on *F*, and *R*- factors based on ALL data will be even larger.

### Fractional atomic coordinates and isotropic or equivalent isotropic displacement parameters (Å^2^)

**Table:**

	*x*	*y*	*z*	*U*_iso_*/*U*_eq_	
S1	0.15719 (4)	0.95193 (10)	0.66141 (3)	0.05013 (15)	
O1	0.18060 (9)	0.6078 (3)	0.54404 (7)	0.0548 (4)	
N1	0.33188 (11)	1.0657 (3)	0.72790 (8)	0.0474 (4)	
C1	0.17643 (14)	1.1896 (4)	0.73578 (10)	0.0449 (5)	
C2	0.11011 (15)	1.3333 (4)	0.76826 (11)	0.0558 (5)	
H2	0.0448	1.3057	0.7506	0.067*	
C3	0.14437 (17)	1.5159 (4)	0.82685 (12)	0.0610 (6)	
H3	0.1015	1.6124	0.8495	0.073*	
C4	0.24200 (17)	1.5602 (4)	0.85322 (11)	0.0593 (5)	
H4	0.2634	1.6883	0.8923	0.071*	
C5	0.30670 (15)	1.4168 (4)	0.82207 (11)	0.0546 (5)	
H5	0.3719	1.4453	0.8402	0.066*	
C6	0.27417 (14)	1.2284 (4)	0.76311 (10)	0.0454 (5)	
C7	0.28134 (13)	0.9108 (4)	0.67424 (10)	0.0420 (4)	
C8	0.32640 (13)	0.7109 (4)	0.63100 (10)	0.0421 (4)	
C9	0.42400 (14)	0.6654 (4)	0.65603 (11)	0.0524 (5)	
H9	0.4582	0.7634	0.6985	0.063*	
C10	0.47127 (14)	0.4785 (4)	0.61944 (12)	0.0596 (6)	
H10	0.5365	0.4496	0.6375	0.071*	
C11	0.42174 (16)	0.3352 (4)	0.55621 (12)	0.0599 (6)	
H11	0.4537	0.2111	0.5308	0.072*	
C12	0.32498 (16)	0.3741 (4)	0.53009 (11)	0.0545 (5)	
H12	0.2917	0.2754	0.4874	0.065*	
C13	0.27700 (13)	0.5594 (4)	0.56711 (10)	0.0453 (4)	
C14	0.12712 (14)	0.4466 (4)	0.48140 (10)	0.0518 (5)	
H14A	0.1321	0.2546	0.4953	0.062*	
H14B	0.1517	0.4722	0.4344	0.062*	
C15	0.02453 (14)	0.5376 (4)	0.46736 (11)	0.0556 (5)	
H15A	−0.0104	0.4565	0.4190	0.067*	
H15B	0.0221	0.7342	0.4607	0.067*	

### Atomic displacement parameters (Å^2^)

**Table:**

	*U*^11^	*U*^22^	*U*^33^	*U*^12^	*U*^13^	*U*^23^
S1	0.0431 (3)	0.0516 (3)	0.0532 (3)	−0.0016 (3)	0.0050 (2)	−0.0062 (2)
O1	0.0427 (8)	0.0624 (9)	0.0539 (8)	0.0000 (6)	−0.0010 (6)	−0.0175 (7)
N1	0.0471 (10)	0.0445 (9)	0.0480 (9)	−0.0022 (8)	0.0044 (7)	−0.0040 (8)
C1	0.0494 (13)	0.0390 (11)	0.0462 (11)	−0.0003 (9)	0.0103 (9)	0.0044 (8)
C2	0.0520 (13)	0.0539 (13)	0.0614 (13)	0.0044 (10)	0.0122 (10)	−0.0004 (10)
C3	0.0730 (16)	0.0560 (14)	0.0575 (12)	0.0126 (11)	0.0215 (10)	−0.0008 (10)
C4	0.0809 (17)	0.0459 (11)	0.0496 (11)	0.0013 (12)	0.0104 (11)	−0.0067 (10)
C5	0.0599 (14)	0.0488 (12)	0.0516 (11)	−0.0042 (10)	0.0041 (9)	−0.0043 (10)
C6	0.0525 (13)	0.0387 (11)	0.0426 (10)	−0.0012 (9)	0.0053 (9)	0.0018 (8)
C7	0.0425 (11)	0.0426 (11)	0.0392 (9)	−0.0024 (9)	0.0053 (8)	0.0031 (8)
C8	0.0421 (12)	0.0410 (11)	0.0421 (10)	−0.0013 (9)	0.0065 (8)	0.0019 (8)
C9	0.0444 (12)	0.0537 (12)	0.0557 (12)	−0.0011 (10)	0.0034 (9)	−0.0036 (9)
C10	0.0443 (12)	0.0659 (15)	0.0670 (13)	0.0061 (11)	0.0087 (10)	−0.0047 (11)
C11	0.0583 (15)	0.0610 (14)	0.0635 (13)	0.0095 (11)	0.0197 (11)	−0.0063 (11)
C12	0.0565 (14)	0.0574 (13)	0.0483 (11)	−0.0001 (10)	0.0087 (9)	−0.0087 (9)
C13	0.0425 (12)	0.0474 (11)	0.0446 (10)	0.0007 (9)	0.0060 (8)	0.0021 (9)
C14	0.0518 (12)	0.0559 (12)	0.0440 (10)	−0.0085 (10)	0.0024 (8)	−0.0065 (9)
C15	0.0510 (13)	0.0652 (13)	0.0452 (11)	−0.0105 (10)	−0.0012 (8)	0.0023 (10)

### Geometric parameters (Å, °)

**Table:**

C1—S1	1.7231 (19)	C9—C10	1.376 (3)
C1—C2	1.398 (3)	C9—H9	0.9300
C1—C6	1.391 (3)	C10—C11	1.371 (3)
C2—C3	1.369 (3)	C10—H10	0.9300
C2—H2	0.9300	C11—C12	1.375 (3)
C3—C4	1.391 (3)	C11—H11	0.9300
C3—H3	0.9300	C12—C13	1.382 (3)
C4—C5	1.367 (3)	C12—H12	0.9300
C4—H4	0.9300	C13—O1	1.373 (2)
C5—C6	1.387 (3)	C14—O1	1.431 (2)
C5—H5	0.9300	C14—C15	1.504 (3)
C6—N1	1.385 (2)	C14—H14A	0.9700
C7—S1	1.7552 (18)	C14—H14B	0.9700
C7—N1	1.299 (2)	C15—C15^i^	1.511 (4)
C7—C8	1.469 (2)	C15—H15A	0.9700
C8—C9	1.390 (3)	C15—H15B	0.9700
C8—C13	1.399 (2)		
			
C6—C1—C2	120.89 (18)	C11—C10—C9	119.68 (19)
C6—C1—S1	109.65 (14)	C11—C10—H10	120.2
C2—C1—S1	129.46 (16)	C9—C10—H10	120.2
C3—C2—C1	117.9 (2)	C10—C11—C12	120.33 (19)
C3—C2—H2	121.0	C10—C11—H11	119.8
C1—C2—H2	121.0	C12—C11—H11	119.8
C2—C3—C4	121.45 (19)	C11—C12—C13	120.22 (19)
C2—C3—H3	119.3	C11—C12—H12	119.9
C4—C3—H3	119.3	C13—C12—H12	119.9
C5—C4—C3	120.5 (2)	O1—C13—C12	123.13 (17)
C5—C4—H4	119.8	O1—C13—C8	116.46 (16)
C3—C4—H4	119.8	C12—C13—C8	120.41 (18)
C4—C5—C6	119.4 (2)	O1—C14—C15	107.66 (15)
C4—C5—H5	120.3	O1—C14—H14A	110.2
C6—C5—H5	120.3	C15—C14—H14A	110.2
N1—C6—C5	125.23 (18)	O1—C14—H14B	110.2
N1—C6—C1	114.94 (16)	C15—C14—H14B	110.2
C5—C6—C1	119.82 (18)	H14A—C14—H14B	108.5
N1—C7—C8	121.55 (17)	C14—C15—C15^i^	113.7 (2)
N1—C7—S1	114.57 (13)	C14—C15—H15A	108.8
C8—C7—S1	123.83 (13)	C15^i^—C15—H15A	108.8
C9—C8—C13	117.81 (17)	C14—C15—H15B	108.8
C9—C8—C7	117.97 (16)	C15^i^—C15—H15B	108.8
C13—C8—C7	124.20 (17)	H15A—C15—H15B	107.7
C10—C9—C8	121.54 (18)	C7—N1—C6	111.40 (16)
C10—C9—H9	119.2	C13—O1—C14	117.86 (14)
C8—C9—H9	119.2	C1—S1—C7	89.39 (9)
			
C6—C1—C2—C3	0.9 (3)	C10—C11—C12—C13	0.4 (3)
S1—C1—C2—C3	−179.08 (15)	C11—C12—C13—O1	−179.97 (18)
C1—C2—C3—C4	0.5 (3)	C11—C12—C13—C8	0.6 (3)
C2—C3—C4—C5	−1.4 (3)	C9—C8—C13—O1	179.64 (16)
C3—C4—C5—C6	0.8 (3)	C7—C8—C13—O1	0.9 (3)
C4—C5—C6—N1	−178.41 (18)	C9—C8—C13—C12	−0.9 (3)
C4—C5—C6—C1	0.7 (3)	C7—C8—C13—C12	−179.58 (17)
C2—C1—C6—N1	177.62 (16)	O1—C14—C15—C15^i^	−69.4 (3)
S1—C1—C6—N1	−2.4 (2)	C8—C7—N1—C6	−177.23 (15)
C2—C1—C6—C5	−1.6 (3)	S1—C7—N1—C6	0.4 (2)
S1—C1—C6—C5	178.43 (14)	C5—C6—N1—C7	−179.52 (17)
N1—C7—C8—C9	8.4 (3)	C1—C6—N1—C7	1.3 (2)
S1—C7—C8—C9	−168.92 (14)	C12—C13—O1—C14	3.6 (3)
N1—C7—C8—C13	−172.87 (18)	C8—C13—O1—C14	−176.95 (16)
S1—C7—C8—C13	9.8 (2)	C15—C14—O1—C13	179.99 (15)
C13—C8—C9—C10	0.2 (3)	C6—C1—S1—C7	2.04 (14)
C7—C8—C9—C10	179.00 (18)	C2—C1—S1—C7	−177.94 (18)
C8—C9—C10—C11	0.8 (3)	N1—C7—S1—C1	−1.44 (14)
C9—C10—C11—C12	−1.1 (3)	C8—C7—S1—C1	176.08 (15)

Symmetry codes: (i) −*x*, −*y*+1, −*z*+1.

### Table 1 The observed 'π···π' interaction distances (Å) for the title compound.

**Table:**

Cg-Cg^i^	d_centroids_	d_perpendicular_
Cg1-Cg2_i_	3.775 (11)	3.515
Cg1-Cg3_i_	3.7934 (12)	3.59

Cg1, Cg2 and Cg3 are the centroids of atoms S1/N1/C1/C6/C7, (C1–C6) and (C8–C13) rings, respectively. Symmetry code: (i) x, 1+y, z
